# A Role of *SCN9A* in Human Epilepsies, As a Cause of Febrile Seizures and As a Potential Modifier of Dravet Syndrome

**DOI:** 10.1371/journal.pgen.1000649

**Published:** 2009-09-18

**Authors:** Nanda A. Singh, Chris Pappas, E. Jill Dahle, Lieve R. F. Claes, Timothy H. Pruess, Peter De Jonghe, Joel Thompson, Missy Dixon, Christina Gurnett, Andy Peiffer, H. Steve White, Francis Filloux, Mark F. Leppert

**Affiliations:** 1 Department of Human Genetics, University of Utah, Salt Lake City, Utah, United States of America; 2 VIB Department of Molecular Genetics, University of Antwerp, Antwerp, Belgium; 3 Department of Pharmacology and Toxicology, Anticonvulsant Drug Development Program, University of Utah, Salt Lake City, Utah, United States of America; 4 Division of Pediatric Neurology, University of Utah, Salt Lake City, Utah, United States of America; 5 Department of Neurology, Washington University School of Medicine, St. Louis, Missouri, United States of America; 6 Division of Medical Genetics, University of Utah, Salt Lake City, Utah, United States of America; The Jackson Laboratory, United States of America

## Abstract

A follow-up study of a large Utah family with significant linkage to chromosome 2q24 led us to identify a new febrile seizure (FS) gene, *SCN9A* encoding Na_v_1.7. In 21 affected members, we uncovered a potential mutation in a highly conserved amino acid, p.N641Y, in the large cytoplasmic loop between transmembrane domains I and II that was absent from 586 ethnically matched population control chromosomes. To establish a functional role for this mutation in seizure susceptibility, we introduced the orthologous mutation into the murine *Scn9a* ortholog using targeted homologous recombination. Compared to wild-type mice, homozygous *Scn9a* ^N641Y/N641Y^ knockin mice exhibit significantly reduced thresholds to electrically induced clonic and tonic-clonic seizures, and increased corneal kindling acquisition rates. Together, these data strongly support the *SCN9A* p.N641Y mutation as disease-causing in this family. To confirm the role of *SCN9A* in FS, we analyzed a collection of 92 unrelated FS patients and identified additional highly conserved Na_v_1.7 missense variants in 5% of the patients. After one of these children with FS later developed Dravet syndrome (severe myoclonic epilepsy of infancy), we sequenced the *SCN1A* gene, a gene known to be associated with Dravet syndrome, and identified a heterozygous frameshift mutation. Subsequent analysis of 109 Dravet syndrome patients yielded nine Na_v_1.7 missense variants (8% of the patients), all in highly conserved amino acids. Six of these Dravet syndrome patients with *SCN9A* missense variants also harbored either missense or splice site *SCN1A* mutations and three had no *SCN1A* mutations. This study provides evidence for a role of *SCN9A* in human epilepsies, both as a cause of FS and as a partner with *SCN1A* mutations.

## Author Summary

Febrile seizures are the most common seizure disorder of early childhood, and exhibit a prevalence of 2%–5% in European and North American children. While the genetic basis of febrile seizures is well-documented, efforts to uncover these genes have yielded only a few genes in a small proportion of cases. In a genomic region on human chromosome 2 known to harbor the febrile seizure *SCN1A* sodium channel gene, we now report a disease-causing mutation in the adjacent gene, *SCN9A* (Na_v_1.7), in a large family with febrile seizures. We introduced the family mutation (N641Y) into the orthologous mouse gene to create a knockin mouse model, and tested seizure susceptibility in these mice. Compared to wild-type mice, our *Scn9a* knockin mice have a significantly lower threshold to electrically induced seizures and experience seizures at a significantly faster rate with repeated subthreshold stimulation. We also report novel missense *SCN9A* mutations in unrelated febrile seizure patients. Furthermore, we show that a subset of patients with the catastrophic early-onset Dravet syndrome who commonly have mutations in *SCN1A* also harbor mutations in *SCN9A*. This finding is important as it demonstrates for the first time mutational evidence for a modifying digenic mechanism of human epilepsy. For infants with Dravet syndrome, a genetic diagnosis will be of immediate benefit to guide therapeutics away from the sodium channel blocking class of anticonvulsant drugs that exacerbate seizures but are often the first administered.

## Introduction

Febrile seizures (FS) are the most common seizure disorder of early childhood, and exhibit a prevalence of 2–5% in European and North American children [1]. Large FS families reported in the clinical literature support a genetic etiology for febrile seizures, as does the 31% incidence of FS in first-degree relatives [2]. Individuals who experience FS have a 2–9% chance of developing afebrile seizures later in life [1] and this incidence is four times higher if there is a family history of FS [3]. These later-onset epileptic phenomena include generalized convulsive, as well as simple and complex partial seizures that can be resistant to currently available anticonvulsant therapy [1]. Notably, FS occur in up to 75% of children with the catastrophic early-onset epilepsy disorder of ‘severe myoclonic epilepsy of infancy’ or Dravet syndrome. In Dravet syndrome, a normally developing child at 2 to12 months of age has convulsive seizures that are prolonged and indiscriminately lateralized. In the second or third year of life, these children have frequent myoclonic, partial and atypical absences as well. While the myoclonic attacks disappear after 4–7 years, these children continue to have generalized tonic-clonic, clonic and complex partial seizures with common episodes of nonconvulsive status epilepticus [1].

The hypothesis that alleles predisposing to FS may be found in Dravet syndrome patients led Claes et al. to find a high frequency of *SCN1A* gene mutations in patients with Dravet syndrome [1],[4],[5]. Since then, others have proposed a complex genetic etiology for Dravet syndrome. This is based on the observation that over 50% of Dravet syndrome patients have *de novo SCN1A* mutations yet belong to families with a history of FS [6]–[9]. In addition, identical *SCN1A* missense or truncation mutations are associated with widely different seizure severities, including intractable seizures of Dravet syndrome, comparatively benign FS, and even asymptomatic family members in some cases [10]–[12]. However, definitive genetic evidence supporting a multifactorial hypothesis of Dravet syndrome is lacking [8],[13].

We previously reported linkage (LOD = 8.1) to a 10 centimorgan (cM) region on chromosome 2q24 in a large Utah kindred (K4425) with FS before the age of six years in 21 individuals, including 10 individuals with subsequent afebrile seizures [14]. This region contains five sodium channel α subunit genes including *SCN1A*, *SCN2A* and *SCN3A* that share over 85% identity and are highly expressed in brain [15]. *SCN1A* is commonly mutated in Dravet syndrome and mutations in either *SCN2A* or *SCN1A* are associated with the generalized (genetic) epilepsy febrile seizure plus (GEFS+) syndrome [4], [16]–[18]. Mutations in *SCN2A* have also been reported in patients with benign familial neonatal-infantile seizures and a single *SCN3A* mutation has recently been identified in a pediatric patient with partial epilepsy [19],[20]. Clearly, this genomic region contains several important genes that impact seizure susceptibility in children.

## Results

### Identification of a Familial Mutation in the *SCN9A* Sodium Channel Alpha Subunit Gene

Sequence analysis of whole blood DNA from affected individuals or a monosomal hybrid cell line DNA containing the disease chromosome from patient III-26 in K4425 did not reveal any disease-causing variants within the coding region or exon-intron junctions in either *SCN1A*, *SCN2A*, *SCN3A*, *SCN7A*, *KCNH7 or SLC4A10*, all of which are candidate genes within the linkage region. Deletion/duplication analysis of the *SCN1A* coding region using the multiplex amplicon quantification method [21] in two severely affected K4425 individuals (III-14 and IV-9) was also negative. Furthermore, copy number variation (CNV) analysis of the distal 10 Mb (84%) K4425 linkage region, including *SCN1A*, *SCN2A*, *SCN3A*, *SCN7A* and *SCN9A*, ascertained by using the Agilent array comparative genomic hybridization platform, found no shared CNV between two affected K4425 individuals, III-12 and IV-9 (data not shown). DNA sequencing of five recently identified 5′ UTR exons and seven *cis*-conserved noncoding sequences that overlap two additional 5′UTR exons of *SCN1A* [22],[23] revealed no variants in K4425 III-14 when compared to the reference sequence. Despite extensive analysis of these candidate genes in the linkage region, we were unable to find any disease-causing mutations.

**Figure 1:**
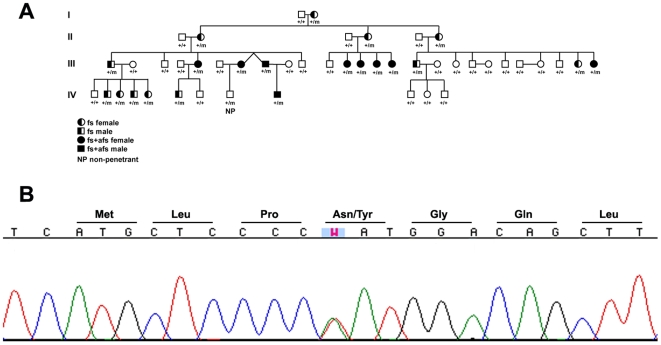
Pedigree of family K4425 with an *SCN9A* mutation. (A) Segregation of the Na_v_1.7 p.N641Y mutation and phenotypic findings of K4425; fs, febrile seizures; afs, afebrile seizures; +, wild-type; m, p.N641Y mutation. (B) Sequence chromatogram of genomic DNA from individual III-1 shows a heterozygous c1921A>T (p.N641Y) mutation in exon 11 of *SCN9A*.

*SCN9A*, which also resides within the K4425 critical genetic interval [14], is expressed primarily in neurons of the dorsal root ganglia and has preliminarily been classified as a peripheral nervous system channel [15]. This expression pattern is consistent with the phenotypes of three inherited disorders that are caused by recently described disease-associated *SCN9A* mutations: autosomal dominant primary erythermalgia (PE) and paroxysmal extreme pain disorder (PEPD), and autosomal recessive channelopathy associated insensitivity to pain (CIP) [24]–[26]. However, early *SCN9A* gene cloning papers [27] showed expression of Na_V_1.7 in brain of rodents and more recent expression analysis experiments have confirmed these observations [28]. The GEO (GDS423 and GDS1085, for example) and Unigene (Hs.439145) databases also contain experimental evidence that SCN9A is expressed in brain. We therefore sequenced all 26 coding exons of *SCN9A* (NM 002977, NP 002968) to test the hypothesis that it harbors the disease-causing allele in FS affected individuals of K4425. Analysis of the large intracellular loop between domains I and II revealed a heterozygous highly conserved missense change (p.N641Y, c.1921A>T) that cosegregates with all 21 affected K4425 individuals, in addition to a single non-penetrant individual (IV-8) (Figure 1A and 1B). A non-penetrant individual is not unexpected as they are commonly seen in autosomal dominant diseases and are well documented in FS pedigrees. Inherited autosomal dominant forms of FS have a reduced penetrance of 60–80% [29]–[31], meaning that 20–40% of individuals with mutations who belong to FS families will not experience seizures. The penetrance of FS in K4425 is actually rather high at approximately 95%. The N641Y variant was absent from 586 chromosomes from an ethnically matched population of unrelated individuals, providing supporting evidence for this nucleotide change being the disease-causing mutation in this family.

### Broad Clinical Spectrum of Seizures in K4425 Individuals with *SCN9A*-N641Y

A broad spectrum of seizure manifestations is observed in K4425 family members who harbor the p.N641Y mutation [14]. Illustrating the milder end of the continuum are 11 individuals from K4425 who experienced only FS before six years of age. The remaining ten of the 21 affected individuals in K4425 experienced FS before six years of age followed by later afebrile seizures. In eight of these ten, the seizures remitted by the age of 16. Finally, two individuals, III-14 and IV-9, developed intractable epilepsy. Patient III-14 experienced her first simple FS at age 1.5 years followed before age five by several non-febrile convulsions and at least one prolonged generalized convulsive seizure lasting at least 45 minutes. After age five, she had occasional complex-partial seizures and was diagnosed with left mesial temporal sclerosis at 22 years of age. At about one year of age, patient IV-9 began having frequent simple FS without focal onset and never lasting more than 2 minutes. However, he had as many as 60 such seizures until about 4–5 years of age. Afebrile generalized convulsive seizures began at about 6 years of age followed closely by very frequent typical absence seizures. He has never had prolonged convulsions, hemiclonic or secondarily generalized seizures, drop attacks, myoclonic or astatic seizures, or “atypical absence” episodes, and there has been no developmental regression. Now 11 years of age, he ultimately has become seizure free with the vagal nerve stimulator (placed at 8 years of age). Electroencephalography demonstrated very frequent frontally predominant generalized 3 to 5 Hz spike and slow wave and polyspike and wave discharges (data not shown). In both of these severely affected K4425 patients, we ruled out additional modifying mutations in four other known FS susceptibility genes, *SCN1A*, *SCN2A*, *SCN1B* and *GABRG2*, by sequencing the coding and splice site regions.

### Reduced Electrical Seizure Thresholds and Increased Corneal Kindling Acquisition Rates in *Scn9a*-N641Y Knockin Mice

**Figure 2:**
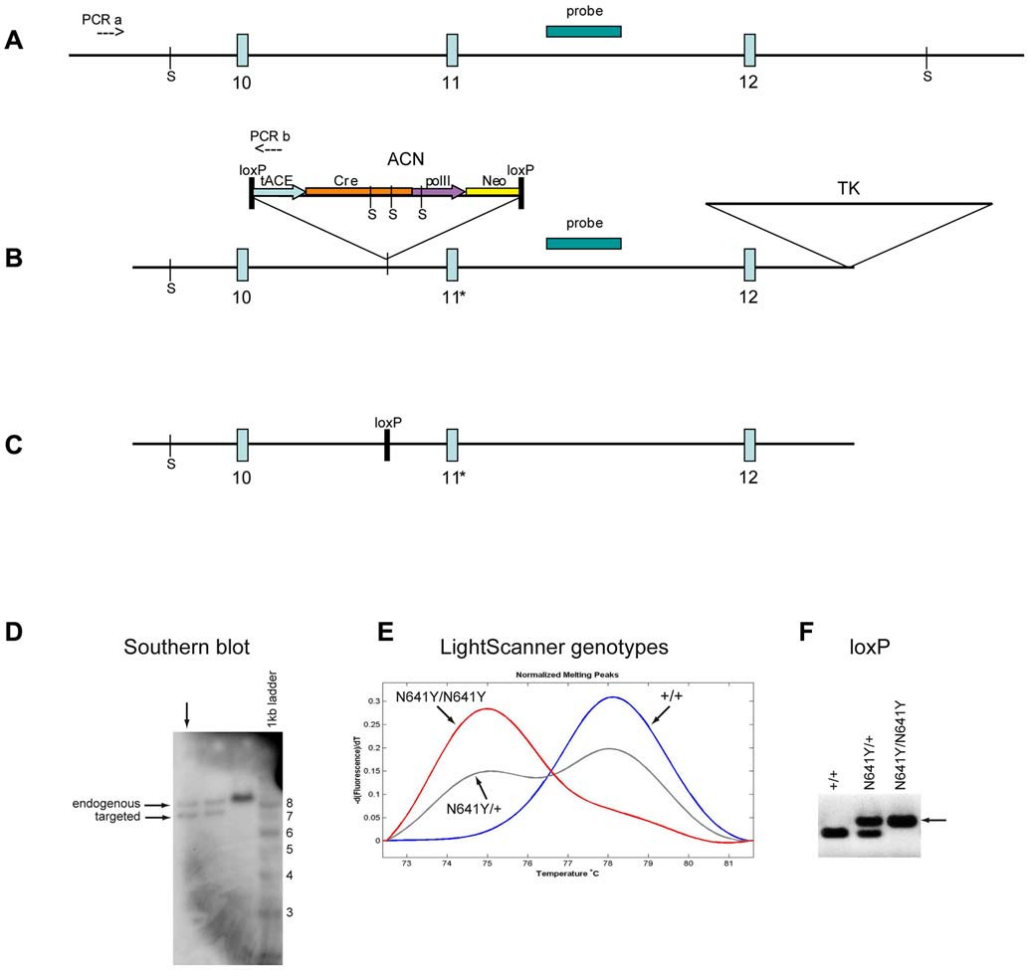
Generation of *Scn9a*-N641Y knockin mice. Schematic representation of the (A) wild-type allele, (B) targeting construct introduced into embryonic stem (ES) cells. Numbered boxes denote exons; *, p.N641Y missense change introduced into exon 11; PCRa and PCRb, primers used to screen ES cell DNA for homologous recombination; S and probe, denotes SspI sites and probe used in genomic Southern blot of ES cells; ACN cassette, Cre-recombinase gene (*Cre*) driven by the testes-specific promoter from the angiotensin-converting enzyme gene (*tACE*); *Cre* is linked to the *Neo* ^r^ selectable marker driven by the mouse RNA polymerase II large subunit gene (*polII*); the entire cassette is flanked by 34 bp loxP sites oriented in parallel. TK, HSV-TK gene for negative selection of ES cells. (C) following *Cre*-mediated self-excision in the chimeric mouse germline, a single loxP site and the point mutation remain. (D) Southern blot of three SspI cut ES cell clones followed by hybridization of probe yields an 8.4 kb endogenous band and a 7.2 kb targeted band (horizontal arrows); vertical arrow denotes clone used to make mouse. (E) LightScanner normalized melting peaks used to genotype *SCN9A* ^+/+^, *SCN9A* ^N641Y/+^, and *SCN9A* ^N641Y/N641Y^ mice. (F) PCR used to verify self-excision of the ACN cassette. Amplicons generated by primers flanking remaining 34 bp loxP site in intron 10 yield distinct *SCN9A* ^+/+^ (left), *SCN9A* ^N641Y/+^ (center), and *SCN9A* ^N641Y/N641Y^ (right, denoted by arrow) bands on 2% agarose.

To confirm the role of *SCN9A* in seizure susceptibility in K4425, we evaluated knockin mice to determine whether the p.N641Y mutation confers a reduced threshold to electrically induced seizures and an enhanced susceptibility to stimulus evoked kindling. Targeted knockin mice were made using previously described methods [32],[33]. Briefly, the following changes were introduced into the wild-type *Scn9a* (Figure 2A): the p.N641Y mutation into exon 11, the ACN positive selection vector into intron 10, and the negative TK selection vector into intron 12 (Figure 2B). Embryonic stem cells evaluated by PCR and Southern blotting techniques were successfully targeted by homologous recombination (Figure 2D). During spermatogenesis, a single male chimera self-excised the positive selection vector (Figure 2C) and at the appropriate age, was mated to a C57BL/6J (B6) female. Genotype analysis reveal that N1F2 offspring had the p.N641Y mutation (Figure 2E) and a single remaining loxP site (Figure 2F). At birth, mutant N1F2 mice showed no significant deviation from Mendelian ratios. The wild-type (B6;129-*Scn9a* ^+/+^)∶heterozygote (B6;129-*Scn9a* ^N641Y/+^)∶homozygote (B6;129-*Scn9a* ^N641Y/N641Y^) birth rate is 105∶182∶101 (*p* = 0.84). No body size difference was observed among the genotypes for either gender and no premature postnatal death occurred up to P90.

**Figure 3:**
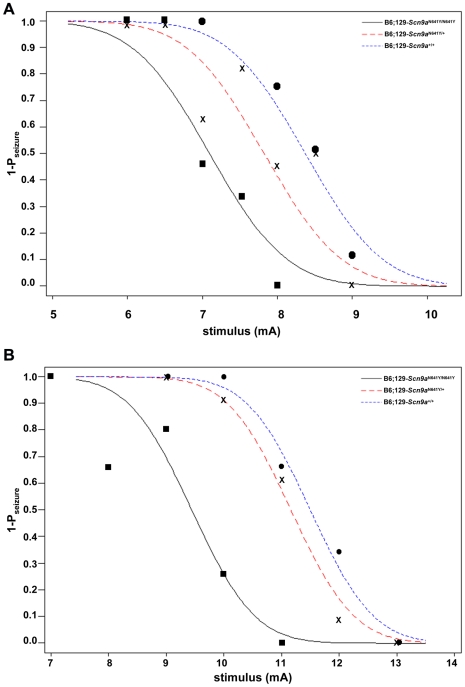
Reduced electroconvulsive seizure thresholds of *Scn9a* knockin mice compared to wild-type littermate controls. Convulsive current curves generated by testing (A) male B6;129-*Scn9a* ^N641Y/N641Y^, B6;129-*Scn9a* ^N641Y/+^, and B6;129-*Scn9a* ^+/+^ mice to minimal clonus electroconvulsive seizures (B6;129-*Scn9a* ^N641Y/N641Y^ vs B6;129-*Scn9a* ^N641Y/+^ *p* = 0.008; B6;129-*Scn9a* ^N641Y/N641Y^ vs B6;129-*Scn9a* ^+/+^ *p* = 0.001; B6;129-*Scn9a* ^N641Y/+^ vs B6;129-*Scn9a* ^+/+^ *p* = 0.093) and (B) female B6;129-*Scn9a* ^N641Y/N641Y^, B6;129-*Scn9a* ^N641Y/+^, and B6;129-*Scn9a* ^+/+^ mice to minimal tonic hindlimb extension electroconvulsive seizures (B6;129-*Scn9a* ^N641Y/N641Y^ vs B6;129-*Scn9a* ^N641Y/+^ *p*<0.001; B6;129-*Scn9a* ^N641Y/N641Y^ vs B6;129-*Scn9a* ^+/+^ *p*<0.001; B6;129-*Scn9a* ^N641Y/+^ vs B6;129-*Scn9a* ^+/+^ *p* = 0.227). Convulsive current data are expressed in terms of 1-seizure probability (1-P_seizure_) for a given stimulus (mA). Individual data points shown for homozygote (closed square), heterozygote (x), and wild-type (closed circle) mice are used to construct curves indicated by black solid, red dashed, and blue dotted lines, respectively.

N1F2 P25–P47 *Scn9a* knockin littermate mice were subjected to corneal electrical stimulation using the staircase method to either a clonic seizure endpoint or tonic hindlimb extension seizure endpoint that depolarizes the forebrain and hindbrain regions, respectively [34]. Convulsive current (CC) levels at which 50% of mice are predicted to seize and the corresponding 95% confidence intervals were calculated for each seizure endpoint. Homozygous B6;129- *Scn9a* ^N641Y/N641Y^ knockin mice exhibited significantly reduced thresholds to minimal clonic (Figure 3A) and minimal tonic hindlimb extension (Figure 3B) seizures relative to their wild-type littermates. Figure 3A shows convulsive current curves generated by testing male B6;129-*Scn9a* ^N641Y/N641Y^, B6;129-*Scn9a* ^N641Y/+^ and B6;129-*Scn9a* ^+/+^ mice to minimal clonic electroconvulsive seizures. The CC_50_ value for this type of seizure is significantly lower for B6;129-*Scn9a* ^N641Y/N641Y^ (CC_50_, 7.1 mA) mice compared to B6;129-*Scn9a* ^N641Y/+^ (CC_50_, 7.83 mA) and B6;129-*Scn9a* ^+/+^ (CC_50_, 8.38 mA) mice; B6;129-*Scn9a* ^N641Y/N641Y^ vs B6;129-*Scn9a* ^N641Y/+^ *p* = 0.008; B6;129-*Scn9a* ^N641Y/N641Y^ vs B6;129-*Scn9a* ^+/+^ *p* = 0.001; B6;129-*Scn9a* ^N641Y/+^ vs B6;129-*Scn9a* ^+/+^ *p* = 0.093; n = 30–68. Figure 3B shows convulsive current curves generated by testing female B6;129-*Scn9a* ^N641Y/N641Y^, B6;129-*Scn9a* ^N641Y/+^ and B6;129-*Scn9a* ^+/+^ mice to minimal tonic hindlimb extension electroconvulsive seizures. Female B6;129-*Scn9a* ^N641Y/N641Y^ (CC_50_, 9.44 mA) mice exhibited a significantly lower CC_50_ value compared to heterozygous B6;129-*Scn9a* ^N641Y/+^ (CC_50_, 11.16 mA) and wild-type B6;129-*Scn9a* ^+/+^ (CC_50_, 11.50 mA) mice; B6;129-*Scn9a* ^N641Y/N641Y^ vs B6;129-*Scn9a* ^N641Y/+^ *p*<0.001; B6;129-*Scn9a* ^N641Y/N641Y^ vs B6;129-*Scn9a* ^+/+^ *p*<0.001; B6;129-*Scn9a* ^N641Y/+^ vs B6;129-*Scn9a* ^+/+^ *p* = 0.227; n = 26–49.

**Figure 4:**
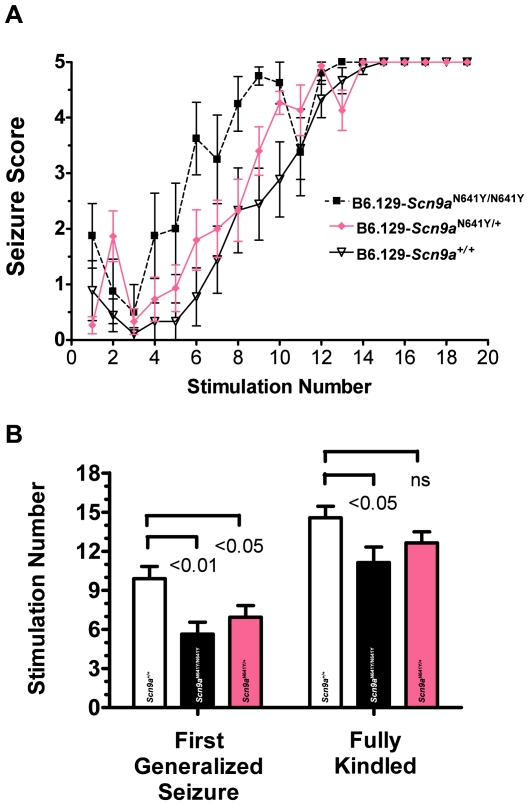
Increased corneal kindling acquisition rates of *Scn9a* knockin mice compared to wild-type littermate controls. Male N5F2 mice separated by genotype (n = 8–15) were stimulated with corneal electrodes twice daily until four consecutive Racine Stage 4 or 5 secondarily generalized seizures were elicited. The effect of *Scn9a*-N641Y on kindling acquisition is shown in (A) for B6.129-Scn9a^+/+^, B6.129-Scn9a^N641Y/+^, and B6.129-Scn9a^N641Y/N641Y^ mice; results are expressed as the average seizure score per genotype observed after each stimulation. (B) The number of stimulations required to reach the first fully generalized Racine Stage 4–5 seizure, regraphed with p-values from the data in (A), is 9.89±0.93 (B6.129-Scn9a^+/+^, clear bar), 5.63±0.92 (B6.129-Scn9a^N641Y/N641Y^, black bar), and 6.93±0.89 (B6.129-Scn9a^N641Y/+^, pink bar), left panel; the number of stimulations required to reach a fully kindled mouse defined as four consecutive Racine Stage 4–5 seizures, regraphed with p-values from the data in (A), is 14.56±0.88 (B6.129-Scn9a^+/+^, clear bar), 11.13±1.2 (B6.129-Scn9a^N641Y/N641Y^, black bar), 12.64±0.86 (B6.129-Scn9a^N641Y/+^, pink bar), right panel.

In a separate test to evaluate seizure susceptibility, the rate of kindling acquisition was evaluated in *Scn9a*-N641Y knockin mice. Male N5F2 P69–P164 *Scn9a* knockin littermate mice were stimulated twice daily with a subthreshold 3mA corneal stimulation for 3 seconds until they reached a stably kindled state, defined as four consecutive secondarily generalized seizures (Racine stage 4–5) [35]. Homozygous B6.129- *Scn9a* ^N641Y/N641Y^ knockin mice exhibited a significantly faster kindling acquisition rate relative to their wild-type littermates (Figure 4A). A significantly lower number of stimulations (Figure 4B) was required to reach the first fully generalized (5.63±0.92, 6.93±0.89, 9.89±0.93) and fourth consecutive generalized (11.13±1.2, 12.64±0.86, 14.56±0.88) seizure for B6.129-*Scn9a* ^N641Y/N641Y^ mice compared to B6.129-*Scn9a* ^N641Y/+^ and B6;129-*Scn9a* ^+/+^ mice, respectively.

Two homozygous mutant female mice were video-monitored continuously from P33–P47 and three homozygous mutant male mice were continuously video-monitored from P27–P50 during the 12-hour daylight cycle. We did not observe any behavior, such as rearing and falling or forelimb or hindlimb clonus, consistent with spontaneous seizures in this time period for any of the mice. Increased ectopic expression of neuropeptide Y in hippocampal dentate granule cell mossy fibers typically indicates spontaneous generalized seizure activity. Increased NPY expression was not seen in P30, P60 and P90 N1F2 B6;129-*Scn9a* ^N641Y/N641Y^ or N1F2 B6;129-*Scn9a* ^N641Y/+^ mice (data not shown). Taken together, these data suggest that *Scn9a*-N641Y knockin mice did not exhibit spontaneous seizures. It is not unexpected that our mouse model of human FS does not exhibit spontaneous seizures because patients with this mutation require hyperthermia for seizures to manifest.

### Expanding the Role of *SCN9A* in Unrelated Febrile Seizure Patients

**Figure 5:**
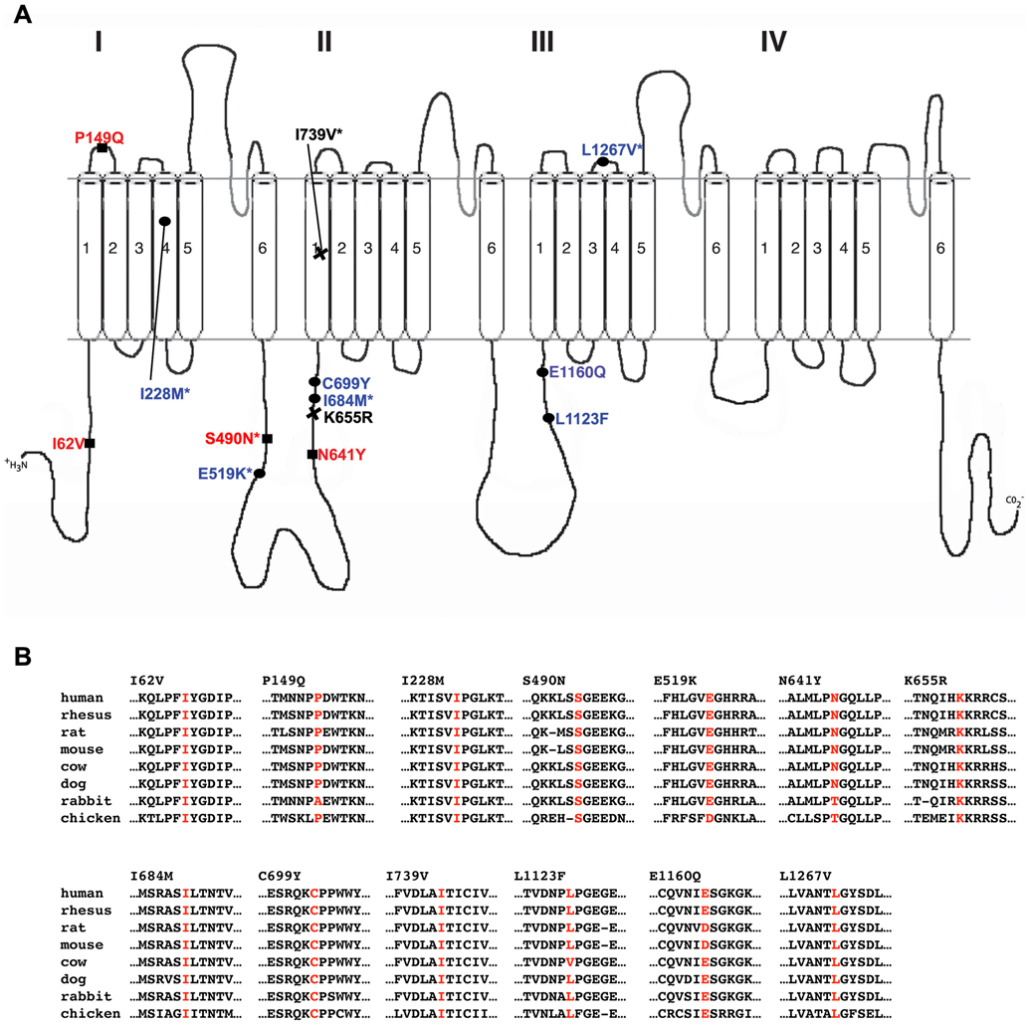
*SCN9A* is mutated in multiple patients with febrile seizures (FS) and Dravet syndrome. (A) Phenotypic profile and secondary structure locations of all variants found in *SCN9A*. Red text, variants in FS patients; blue text, variants in Dravet syndrome patients; black text, variants in both phenotypes; *variants also found in controls. (B) Amino acids from the UCSC genome browser (http://genome.ucsc.edu/) showing conservation across 8 species for FS and Dravet syndrome variants (red) found in *SCN9A*. The human Na_v_1.7 protein shares identity of 97% to rhesus, 92% to rat, 92% to mouse, 94% to cow, 94% to dog, 93% to rabbit, and 75% to chicken.

To further assess the role of Na_v_1.7 in FS patients, we then analyzed *SCN9A* in a panel of 92 unrelated patients with childhood seizures occurring in the setting of a febrile illness, either with or without a family history of seizures. We identified 4 additional missense variants in our 90 Caucasian samples and 1 variant in our 2 Hispanic samples (Figure 5A, Table 1). p.P149Q and p.K655R were not found in at least 562 ethnically matched Caucasian population control chromosomes, while p.S490N and p.I739V were found only once in at least 562 ethnically matched Caucasian population control chromosomes. For all four Caucasian mutations, Fisher's exact two-tailed test yielded *p* = 0.03 (4/180 unrelated FS chromosomes and 2/562 population control chromosomes). The single Hispanic variant p.I62V was not found in 276 ethnically matched Hispanic control chromosomes (*p* = 0.01 for 1/4 FS chromosomes and 0/276 population control chromosomes, Fisher's exact two-tailed test). All five seizure-associated Na_v_1.7 variants reported here occur in codons that are highly conserved across species (Figure 5B). To rule out the role of *SCN1A* in FS susceptibility in these 5 FS patients with *SCN9A* variants, we sequenced the entire coding and splice site regions of *SCN1A* and did not find any potential disease-causing amino acid variations.

**Table 1:** Overview of patients with *SCN9A* variants and their corresponding *SCN1A* mutation.

Sample	Phenotype	*SCN9A* (Inheritance)	*SCN1A* (Inheritance)
K4425 (n = 21)	FS, AFS, TLE	p.N641Y/c.1921A>T (AD)	none
34351	FS	p.I62V/c.184A>G (n.a.)	none
40095	FS	p.P149Q/c.446C>A (n.a.)	none
EPD279.1	complex FS	p.S490N*/c.1469G>A (n.a.)	none
34447	FS, GSW, IGE	p.K655R/c.1964A>G (n.a.)	none
33418	FS, IGE	p.I739V*/c.2215A>G (P)	none
EP272.01	SMEB-MA	p.I228M*/c.684C>G (M)	p.V982L/c.2944G>C (*de novo*)
EPD232.1	Dravet	p.E519K*/c.1555G>A (M)	none
EP268.01	Dravet	p.K655R/c.1964A>G (P)	p.M934I/c.2802G>A (*de novo*)
EPD72.1	Dravet	p.K655R/c.1964A>G (n.a.)	none
EP64.03	Dravet	p.I684M*/c.2052A>G (M)	c.4338+1G>A (*de novo*)
EP260.01	Dravet	p.C699Y/c.2096G>A (P)	c.1029-1G>A (*de novo*)
EP263.01	SMEB-MA	p.I739V*/c.2215A>G (P)	p.A1326D/c.3977C>A (*de novo*)
34302	Dravet	p.L1123F*/c.3369G>T (M)	p.N892fsX2/c.2675delA (*de novo*)
EPD189.1	Dravet	p.E1160Q/c.3478G>C (M)	none
EPD227.1	Dravet	p.L1267V*/c.3799C>G (n.a.)	c.3706-2A>G (n.a.)

*: In <0.3% controls. FS, febrile seizures; AFS, afebrile seizures; TLE, temporal lobe epilepsy; GSW, generalized spike wave; IGE, idiopathic generalized epilepsy; SMEB-MA, Dravet syndrome without myoclonic seizures and ataxia.

Reference sequences used are: SCN9A (NP 002968) and SCN1A (Swiss-Prot P35498).

AD, autosomal dominant; P, paternal; M, maternal; n.a., parents not available.

### A Role for *SCN9A* in Dravet Syndrome

**Figure 6:**
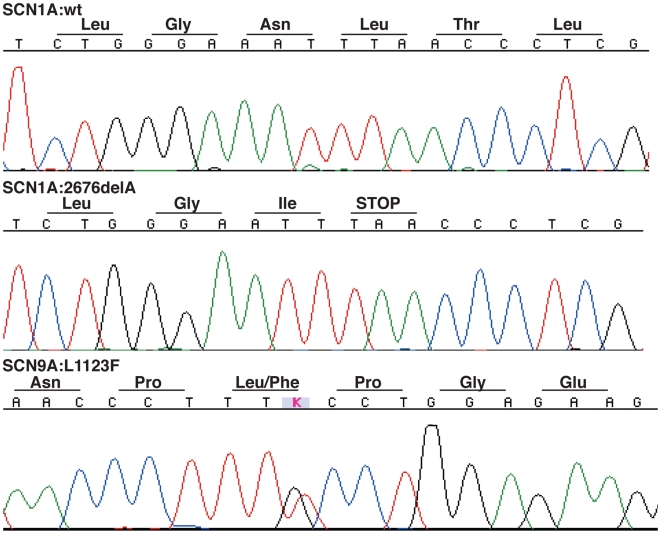
Utah Dravet syndrome patient #34302 harbors mutations in both *SCN9A* and *SCN1A*. Sequence chromatograms of wild-type (top panel) and mutant (middle panel) clones of *SCN1A* exon 15 reveals a frameshift mutation p.N892fsX2 (c.2675delA); sequence chromatogram of genomic DNA shows a heterozygous p.L1123F (c.3369G>T) in exon 17 of *SCN9A* exon (bottom panel).

During the course of our studies on unrelated FS patients, the diagnosis of one patient #34302 progressed from atypical FS to Dravet syndrome. Beginning at five months of age, this Caucasian patient experienced multiple generalized clonic seizures that were predominantly afebrile, then progressed to frequent episodes of status epilepticus and prolonged complex partial seizures by 16 months. Now 5 years old, this patient continues to have mixed seizures (including myoclonic and astatic seizures) in spite of resolute therapeutic intervention. Sequencing of *SCN9A* yielded a p.L1123F missense variant found only once in 1736 ethnically matched population control chromosomes (Fisher's exact *p*-value = 0.0023; Figure 6, Table 1). Segregation analysis showed that the *SCN9A* p.L1123F variant was inherited from the asymptomatic mother with a reported extended family history of seizures. Subsequent sequencing of the *SCN1A* gene (Swiss-Prot P35498) known to cause Dravet syndrome uncovered a heterozygous frameshift mutation (c.2675delA, p.N892fsX2) in the intracellular loop between DIIS4 and DIIS5 (Figure 6, Table 1). The *SCN1A* frameshift was *de novo* and misinheritance was ruled out by testing 31 polymorphic microsatellite markers (data not shown).

The finding of variants in both *SCN1A* and *SCN9A* in a single patient led us to investigate whether additional disease-associated alleles in *SCN9A* contribute to Dravet syndrome. In an analysis of a cohort of 109 Dravet syndrome patients, 50% of whom had *SCN1A* mutations, we found 8 additional *SCN9A* variants within the transmembrane domains and intracellular and extracellular loops of Na_v_1.7 in 9 patients (Table 1). The missense variants p.C699Y, K655R and p.E1160Q were not found in at least 576 control chromosomes and the remaining 5 missense variants were found in 0.3% of at least 576 control chromosomes (*p* = 0.004 for 9/218 Dravet syndrome cases and 5/576 population controls, Fisher's exact two-tailed test). Of the 9 Dravet syndrome patients with *SCN9A* variants, six harbor either splice site or missense mutations in *SCN1A* (Table 1, Figure 5). Two of these SCN9A variants (p.K655R and p.I739V) are also found in our FS patients. In the three remaining Dravet syndrome patients without *SCN1A* mutations, additional proconvulsive genes that act in concert with *SCN9A* may yet be uncovered. Protein secondary structure prediction using Consensus Data Mining [36] found coil to α-helix (p.I684M), α-helix to coil (p.I739V, p.L1123F, p.L1267V) and ß-sheet to coil (p.E1160Q) alterations.

An alternate statistical approach is to examine the mutational burden of *SCN9A*, comparing rare (<1%) variants identified by mutational analysis of the entire coding region in all FS and Dravet syndrome populations combined versus the entire coding region in population control individuals. Analysis of the coding and splice site regions of *SCN9A* was performed only in a subset of our control panel consisting of 95 healthy individuals from the Utah CEPH collection and three variants were identified (p.I684M, p.L1267V, p.D1971V). Our affected population in which the entire gene was analyzed consisted of 93 FS including a single person from the family K4425 plus 110 Dravet syndrome patients, including the initial patient #34302. This approach yielded *p*<0.10, Fisher's exact one-tailed test and *p*<0.07, Fisher's Exact-Boschloo with the Berger & Boos correction [37] (3/190 Utah CEPH chromosomes and 16/406 FS and Dravet syndrome unrelated seizure cases combined).

## Discussion

In this study, we have shown that a mutation in a highly conserved amino acid residue of the SCN9A sodium channel alpha subunit is associated with a wide clinical spectrum of seizure phenotypes in a single large family. These phenotypes include simple FS, self-limited afebrile seizures, and temporal lobe epilepsy. The *SCN9A*-N641Y segregating mutation in our large K4425 FS family, and the significantly reduced seizure threshold and enhanced kindling acquisiton rate phenotypes conferred uniquely by the same mutation introduced into the *Scn9a*-N641Y knockin mouse, provide strong evidence that *SCN9A* has a role in central excitability and is disease-causing in this family.

In addition, we find supporting evidence for a multifactorial etiology of Dravet syndrome by uncovering concurrent variants in both *SCN9A* and *SCN1A* in a subset of our patients. Our findings of numerous variants in separate FS and Dravet syndrome cohorts are statistically significant (p<0.05) when the frequency of the combined specific altered residues found in patients is compared to those same residues in controls, but not statistically significant when all variants found in FS and Dravet syndrome patients are compared to all variants found in controls. While our findings provide highly suggestive evidence of the role of *SCN9A* in FS and Dravet syndrome, replication in multiple cohorts, combined with functional studies, is needed to confirm a hyperexcitable role of *SCN9A* in unrelated epilepsy patients. The multifactorial etiology of Dravet syndrome proposed by many investigators suggests that it is very likely that genes responsible for Dravet syndrome will far outnumber *SCN1A* and *SCN9A*.

The corneal stimulation paradigm is a reliable and reproducible measure for inducing seizures to test the efficacy of anticonvulsant drugs [34]. A *Kcnq3* and two separate *Kcnq2* mouse models as well as the models for the Wolf-Hirschhorn deletion syndrome have helped to validate the corneal stimulation paradigm as a robust seizure susceptibility test [33],[38],[39]. Here, we show that homozygous knockin *Scn9a* ^N641Y/N641Y^ mice are significantly more susceptible than wild-type mice to seizures that activate either the forebrain or hindbrain. Both the clonic and tonic-clonic generalized seizures characteristic of FS patients are induced by lower stimulation currents in mice that harbor the p.N641Y mutation in *Scn9a*. In the corneal kindling model, repeated application of an initially subconvulsive electrical stimulus results in progressive escalation of the stimulus-induced epileptic activity, culminating in a partial seizure that secondarily generalizes [35]. This model of partial epilepsy successfully validated a first-in-class neurotherapeutic agent based on galanin for treating pharmacoresistant epilepsy [40] and differentiated knockin mutations known to cause childhood epilepsy. Indeed, mutations in the *Kcnq2* and *Kcnq3* subunits that underlie M-current channels were recently shown to significantly increase the rate of corneal kindling [41]. Our model adds to the growing list of other specific human epilepsy knockin mice, including the *Gabrg2* [42], *Kcnq2* [33], *Kcnq3* [33], *Scn1a* [43] and *Chrna4* [44] mice, to report a clear-cut genotype to phenotype seizure susceptibility.

In multiple published studies, some Dravet syndrome patients inherit *SCN1A* mutations from asymptomatic or mildly affected parents, making multiple mutations in this syndrome a likely finding [7],[11],[12]. Furthermore, modifying alleles may preferentially be found in Dravet syndrome patients with *SCN1A* mutations that are less deleterious when compared to complete heterozygous loss of function mutations. This is indeed that case for the majority of our Dravet syndrome patients with SCN9A variants. Six out of seven of our Dravet syndrome patients with *SCN9A* variants harbor either missense or splice site mutations in *SCN1A* while a sizable portion of published *SCN1A* mutations are predicted to lead to truncated proteins [8]. Our results support the idea that some *SCN9A* variants when found alone might be asymptomatic or cause infrequent febrile seizures due to incomplete penetrance and variable expressivity, but likely contribute in a multifactorial fashion to Dravet syndrome. Indeed, a recent finding of almost 100 unique missense *SCN1A* mutations challenges the previously held notion that haploinsufficient *SCN1A* mutations alone are responsible for Dravet syndrome because many of these missense mutations likely confer only partial, rather than complete, heterozygous loss of function [22]. Our results now suggest that Dravet syndrome may be included in the list of disorders with “modifier” genes that include Huntington's disease and cystic fibrosis [45]. Additional new functional data that examines the two gene mutations will be required to test if the “two-hit” hypothesis is valid in certain Dravet syndrome patients.

None of our FS or Dravet syndrome variants overlaps with the *SCN9A* disease-associated changes found in the extreme pain or insensitivity to pain disorders [24]–[26],[46]. Furthermore, in all published studies of PE, PEPD and CIP, an increased incidence of seizures is not reported in patients with *SCN9A* mutations [24]–[26]. After follow-up questioning, none of the 21 affected members of K4425 reported the easily recognized extreme pain phenotypes associated with some *SCN9A* missense mutations. PEPD is often misdiagnosed as epilepsy because tonic non-epileptic seizures are a particular feature in infancy and early childhood. However the “slow-flat-slow” ictal EEG pattern associated with profound syncope in PEPD patients is clearly not epileptiform, whereas EEGs in K4425 patients are epileptiform [14],[47]. Another distinguishing feature is that PEPD attacks are provoked by physical stimulation and not by hyperthermia as seen in FS.

The notion that dysfunction in the same ion channel can be associated in distinct paroxysmal phenotypes is already known for *SCN9A*. In 17 of 18 patients with *SCN9A* missense mutations published to date, the rectal, ocular and mandibular pain seen in PEPD does not overlap with the severe burning hand and foot pain characteristic of PE [48]. We now extend the tissue specificity of paroxysmal Na_v_1.7 malfunction to the central nervous system. Additional support for discrete phenotypes resulting from the same ion channel protein comes from the identification of *SCN1A* mutations in either epilepsy or familial hemiplegic migraine [49], and *CACNA1A* mutations in familial hemiplegic migraine, episodic ataxia and spinocerebellar ataxia [50]. Experimentally, the Na_v_1.7 p.L858H PE mutation causes hyperexcitable sensory neurons and hypoexcitable sympathetic neurons, and these opposing electrical properties are a result of neuron specific physiologically coupled proteins [51]. A unique complement of Na_v_1.7 interacting proteins or second messenger pathways in the central nervous system may also explain how the same gene previously implicated with peripheral pain can also be associated with an epilepsy phenotype, but this hypothesis will require further study.

## Materials and Methods

### Patient Ascertainment

Institutional Review Board informed consent was obtained from all participants. FS patients in our sample experienced at least one seizure in a setting of fever, concurrent with a visit to the emergency room where the FS diagnosis was first made by the attending physician. The majority, but not all, patients had follow-up care by our epilepsy clinic at the University of Utah, so recurrent FS and the presence of afebrile seizures are known for most patients. Dravet syndrome patients are ascertained by neurologists who follow ILAE clinical criteria and are located primarily throughout Europe.

### Mutation Detection of Patient and Control DNA Samples

DNA isolated from blood (Puregene) was sequenced using primers designed outside the exons of *SCN9A* followed by standard ABI technology at the University of Utah Sequencing Core Facility. Sequence was analyzed using the Sequencher program (Gene Codes Corporation). Mutation detection of control and disease cohorts was done either by sequencing or by the LightScanner system using the manufacturer's recommendations (Idaho Technology). Copy number variation analysis comparing two affected individuals and their respective unaffected parent was performed using the Agilent array comparative genomic hybridization platform. We hybridized 4×44 K arrays at an average resolution of 3 probes per kB, including 5% exonic probes and 56% intragenic probes, in accordance with the manufacturer's specifications. Deletion-duplication analysis was performed using the multiplex amplicon quantification method [21]. Fisher's exact two-tailed test was performed to assess significance.

### Generation of B6.129- *Scn9a* Knockin Mice

Wild-type clones of *Scn9A* were isolated from a mouse BAC clone library (CHORI) and subcloned into pUC18. The p.N641Y point mutation in exon 11 was introduced using the QuickChange II XL system (Stratagene). The ACN cassette was cloned into a PmlI site in intron 10 and this construct was cloned into a thymidine kinase (*TK*) vector [32]. Within the ACN cassette the neomycin (*neo*) gene driven by the mouse RNA polymerase II promoter (*polII*) confers positive selection and the *TK* gene confers negative selection of ES cells. The targeting vector was linearized with NotI, introduced by electroporation into R1 ES cells [52] and selected for resistance to G418 and FIAU. DNA from 104 colonies was isolated and screened for homologous recombination by PCR using primers designed outside the construct and within the ACN cassette. Three positives were sequenced to determine the presence of the mutation. Southern blot analysis was done on three SspI cut ES cell clones to verify presence and orientation of endogenous and targeted alleles. Hybridization of ^32^P-labelled probe in intron 11 yields an 8.4 kb endogenous band and a 7.2 kb targeted band in mutation positive sample. ES cells from this single targeted clone were aggregated with C57BL/6-derived morulae, and implanted into a pseudopregnant C57BL-6 female. During chimeric male spermatogenesis, Cre recombinase (*Cre*) driven by the murine angiotensin-converting enzyme promoter, *tACE*, confers loxP-mediated excision of the ACN cassette to yield a single remaining loxP site. Chimeric progeny were identified by coat color and nine males were crossed to C57BL/6J (Jackson labs) females for the generation of F1 offspring. F1 offspring were intercrossed to generate F2 experimental animals.

To detect *Cre*-mediated self-excision of the ACN cassette and presence of the mutation, genomic DNA obtained from tail biopsies of F1 and F2 animals was analyzed. PCR primers were used to asymmetrically amplify a product containing the mutated base pair. An unlabeled oligonucleotide probe complimentary to the excess strand in the region surrounding the mutated base was included and the reaction melted using a LightScanner (Idaho Technology) instrument. Melt curves were analyzed using LightScanner software (Idaho Technology) and distinct melt profiles were recognizable for each genotype. To verify self-excision, primers surrounding the remaining loxP site were used to amplify PCR products that were electrophoresed on a 2% agarose gel. The presence of a single loxP site verifies self-excision. Mouse colonies were maintained and used experimentally at the University of Utah in accordance with Institutional Animal Care and Use Committee approved protocols.

### Evaluation of Electrical Thresholds and Corneal Kindling Acquisition Rates in *Scn9a* ^+/+^, *Scn9a* ^+/N641Y^, and *Scn9a* ^N641Y/N641Y^ Littermate Mice

For baseline seizure threshold estimates, seizure incidence was determined at several different stimulus intensities according to the staircase estimation procedure [53]. Convulsive current (CC) curves were then constructed from these data by Probit analysis, and CC_1–99_ values statistical comparisons were calculated using Minitab 13 (State College, PA, U.S.A.) and p values are calculated for full CC curve comparisons. CC curves for knockin mice were compared with those of littermate wild-type mice and seizure thresholds were considered significantly different at p<0.05. Two different stimulation protocols were used in an effort to differentiate the effects of genotype on forebrain (minimal clonic) and hindbrain (minimal tonic hindlimb extension) seizure thresholds. Seizures were induced at varying intensities using a 60-Hz, 0.2-ms sinusoidal current pulse with a stimulator previously described [53]. A drop of tetracaine (0.5%) was administered to each eye just before testing. Minimal clonic seizures are characterized by rhythmic face and forelimb clonus, rearing and falling and ventral neck flexion. Minimal tonic hindlimb extension seizures are characterized by a tonic–clonic flexion–extension sequence that starts with tonic forelimb extension, followed by hindlimb flexion, and terminates in full tonic hindlimb extension (180 degrees to the torso) [53].

Individual adult male N5F2 mice were corneally kindled according to the procedures described by Matagne and Klitgaard [35]. Briefly, each mouse received a twice-daily corneal stimulation of 3mA for 3 seconds. Prior to each stimulation, a drop of 0.5% tetracaine was applied to the cornea of each mouse to provide anesthesia and aid electrical conduction. Seizure severity was ranked according to the Racine scale [54]: 1, jaw chomping and vibrissae twitching; 2, facial clonus, head nodding, chewing; 3, unilateral or alternating forelimb clonus; 4, bilateral forelimb clonus with rearing and falling; 5, generalized clonus with immediate loss of balance. The kindling procedure was continued until each mouse displayed at least five consecutive stage 4–5 secondarily generalized seizures. Results obtained for *Scn9a*-N641Y homozygous and heterozygous knockin mice were compared to wild-type littermates and expressed as the average behavioral seizure score observed for each stimulation, the number of stimulations required to reach the first stage 4–5 seizure, and the number of stimulations required to reach four consecutive stage 4–5 seizures, or a fully kindled state.
